# Outcomes and Prognosis of Non-Elderly Patients with Brain Metastases—A Prospective Cohort Incorporating Individualized Assessment of Heart Rate Variability

**DOI:** 10.3390/jpm11111049

**Published:** 2021-10-20

**Authors:** Yu-Ming Wang, Jen-Yu Cheng, Chong-Jong Wang, Shu-Shya Hseu, Eng-Yen Huang

**Affiliations:** 1 Department & Institute of Physiology, National Yang Ming Chiao Tung University, Taipei 11221, Taiwan; scorpion@cgmh.org.tw; 2 Department of Radiation Oncology & Proton and Radiation Therapy Center, Kaohsiung Chang Gung Memorial Hospital, Chang Gung University College of Medicine, Kaohsiung 83301, Taiwan; york480@cgmh.org.tw (J.-Y.C.); cjw1010@cgmh.org.tw (C.-J.W.); 3 School of Traditional Chinese Medicine, Chang Gung University, Taoyuan 33302, Taiwan; 4 Department of Anesthesiology, Taipei Veterans General Hospital, Taipei 11217, Taiwan; sshseu@vghtpe.gov.tw

**Keywords:** brain metastases, non-elderly, age, heart rate variability, radiotherapy, prognosis

## Abstract

Outcomes and prognostic factors among patients with brain metastases (BM) have been widely studied, but seldom for the non-elderly. Heart rate variability (HRV) is a physiological phenomenon and has been shown as a survival prognostic factor in cancer patients. This study aimed to evaluate the outcomes and prognosis among non-elderly BM patients with the incorporation of HRV analysis. Forty non-elderly BM patients treated using whole brain radiotherapy (WBRT) were studied from January 2010 prospectively with 5-min electrocardiography (ECG) recordings. Individualized HRV was generated by the ECG, and the time domain HRV index SDNN was chosen for survival analysis. The median overall survival (OS) for the entire group was 6.21 months. Univariate analysis revealed that a KPS < 80 (*p* = 0.019) and an SDNN < 10 ms (*p* = 0.007) demonstrated statistical significance for OS; multivariate analysis confirmed that a KPS < 80 (*p* = 0.004; HR = 3.060, CI = 1.437–6.517) and an SDNN < 10 ms (*p* = 0.010; HR = 2.664, CI = 1.262–5.624) were independent prognostic factors. Prospective studies for risk stratification among non-elderly BM patients based on our results are warranted to validate our findings.

## 1. Introduction

Brain metastases (BM) frequently occur in patients with advanced cancer, and are the most common intracranial tumor, estimated to be 10 times more frequent than primary brain tumors [1,2]. Although different treatment modalities have been reported, the outcome is still unsatisfactory [3]. Previous large prospective studies demonstrated that age is an independent survival prognostic factor in BM patients. Patients with a younger age have better survival compared to the elderly [4,5,6,7]. With emerging novel modalities of therapeutic options, non-elderly BM patients, especially those with longer life expectancies, should be considered differently and treated more aggressively, as they are more tolerant to treatments [8,9,10]. Nieder et al. found that survival prognostic factors in non-elderly BM patients differ from older patients [11]. Nevertheless, studies focusing on outcomes and prognosis among non-elderly BM patients still need more investigation.

Heart rate variability (HRV) is a physiologic phenomenon that is predominantly regulated by the vagus nerve [12]. It represents the complexity of the physiological system that controls homeostasis. With the standardized method for recording and measuring, HRV is well-documented and can be assessed in clinics non-invasively [13]. Subjects with a lower HRV, which can be a surrogate of impaired vagal function, are at higher risk for cancer-prone environment and with higher risk for life threatening cardio-cerebral circulation events [14,15,16]. In addition to the theoretical background, clinical use of HRV have also been widely studied. The correlation between HRV and the severity of disease, such as acute myocardial infarction, heart failure, diabetes mellitus, and cancer survival, has been published the previous literature. Patients with lower HRV did predict inferior outcomes and survival [17,18,19,20,21]. However, the application of this finding for non-elderly BM patients remains unclear.

To understand and stratify the risk among non-elderly BM patients and to incorporate the novel factor HRV, this study was conducted to further investigate this group of patients.

## 2. Materials and Methods

### 2.1. Participants

This prospective study began in January 2010, and our Institutional Review Board approved all protocols (98-3760B). The recruited BM patients were those who underwent palliative whole brain radiotherapy (WBRT) in our department and agreed to participate in HRV analysis. Patients with a history of central nervous system or thoracic surgery, or with underlying arrhythmia, or actively taking medications that could influence HRV were excluded. Before entering the study, the participants’ clinical history, previous cancer treatments, and radiological factors were recorded; and all participants also underwent a comprehensive physical examination before the start of treatment. An electrocardiogram (ECG) was performed during the initial evaluation for HRV analysis. To assess the clinical impact of HRV in non-elderly patients, defined as being aged under 65 years old, data from patients who met this age definition were used in the following analysis.

### 2.2. ECG Signal Processing for HRV

The HRV analysis protocol has been reported previously [19], summarized as follows. Five-minute digitalized ECG was acquired from all patients on the same day or one day before WBRT simulation in a quiet room in supine position. The R peaks of each valid QRS complex were labelled for processing for HRV using the standardized HRV analytic method [13]. Based on previously published results, the standard deviation of normal-to-normal intervals (SDNN), the global HRV index, was used as a prognostic factor for survival analysis.

### 2.3. Radiotherapy and Follow-Up

As this study protocol was not designed to change the standard care, all patients were treated following our public health insurance policy for BM patients using WBRT, with selected patients receiving an additional local boost (LB). Patients were immobilized with a thermoplastic cast in supine position and simulated by a CT simulator with a 3.75 mm slice thickness. WBRT fields were designed as conventional bilateral opposed helmet fields. Patients were irradiated using 6 or 15 megavolt photons at a dose of 30 to 37.5 Gy in 10 to 15 daily fractions, with 5 fractions per week. Administrating an additional LB to the gross intracranial tumors was suggested for selected patients following WBRT as our routine practice. Generally, patients with good performance whose intracranial tumor were larger than 2 cm in diameter were suggested to receive local boost course. LB was delivered using intensity-modulated radiotherapy for an additional 10 to 14 Gy in 5 to 7 daily fractions targeting only the gross intracranial lesions. After completing the RT course, patients were followed up in a 1-to-2-month intervals. Brain images were performed regularly until their death. For those patients who were not able to be present at our out-patient clinic, telephone contact was used instead.

### 2.4. Study Endpoints and Statistics

The primary endpoint for this study was overall survival (OS); the time of survival was calculated from the day of the ECG recording to the date of death or the last follow-up. All known clinical factors, radiological factors [4,5,6,7], comorbidities such as diabetes mellitus and hypertension, and the HRV index SDNN were used as prognostic factors for the study. IBM SPSS Statistics version 20 (IBM, Armonk, NY, USA) was used for the following calculation. Factors among each group were compared using Chi-square or Fisher’s exact test for categorical variables; an independent *t*-test was used for comparison between continuous variables. OS was analyzed by the Kaplan–Meier method. Prognostic factors were compared using the log-rank test; Cox’s proportional hazard regression method was used for multivariate analysis, and variables that reached *p* < 0.1 in univariate analysis were put into examination. Significant differences were met when *p* < 0.05.

## 3. Results

### 3.1. Characteristics of the Participants

A total of 40 BM patients aged under 65 prospectively recruited into our study were retrospective analyzed. The median age of this cohort was 57, and 60% of these patients were diagnosed with non-small cell carcinoma of the lungs (NSCLC). Most patients had extracranial metastases and did not have the primary origin under control when they were confirmed as having BM. Seventy-five patients had more than three intracranial lesions, and more than 40% of our patients had a volume of the largest intracranial lesion of more than 5 cm^3^. With the relatively young age of these patients, 35% of them had a KPS of 80 or above. For the HRV index, one third had an SDNN < 10 ms. Based on the most used risk stratification system [4], only one patient met the criterion of RPA I (Table 1).

For the following analysis, an SDNN of 10 ms was chosen as the cutoff value [19]. There was no significant difference among each prognostic factor between patients with an SDNN < 10 ms and an SDNN ≥ 10 ms (Table 2).

### 3.2. Treatment Outcomes

**Figure 1:**
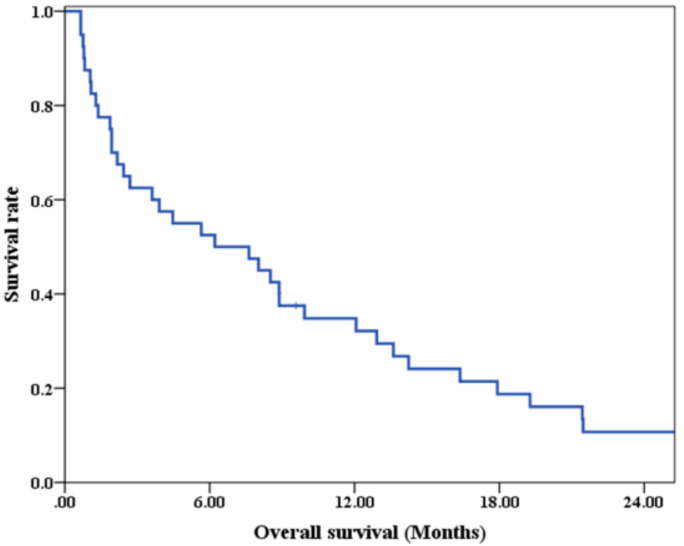
Overall survival of all non-elderly brain metastases patients.

At the time we performed the analysis, three patients were alive. The median follow-up time of these survivors was 49.74 months. The median OS of the entire groups was 6.21 months, with 3-month, 6-month, and 12-month survival of 62.5%, 52.5%, and 34.8%, respectively (Figure 1).

### 3.3. Prognostic Factors

**Figure 2:**
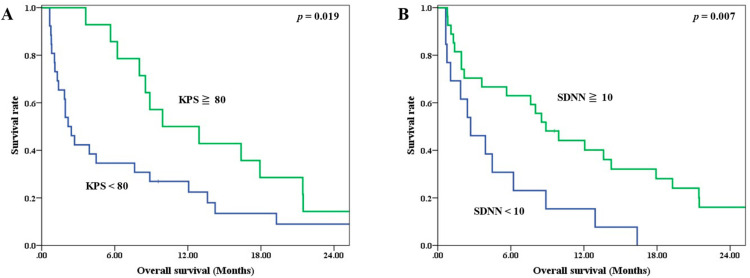
Survival curves stratified by prognostic factors (**A**) KPS, (**B**) SDNN.

Univariate analysis revealed that the KPS and SDNN were significant prognostic factors for OS (Table 3). The median OS for patients with a KPS < 80 and ≧80 were 2.17 and 9.93 months, respectively (*p* = 0.019) (Figure 2A). The corresponding median OS for patients with an SDNN < 10 ms and ≧10 ms were 2.70 and 8.88 months (*p* = 0.007) (Figure 2B).

For multivariate analysis, a KPS of 80 (*p* = 0.018; HR = 2.349, CI = 1.159–4.762) and an SDNN of 10 ms (*p* = 0.008; HR = 2.766, CI = 1.306–5.857) were confirmed as independent OS prognostic factors (Table 4). Factors other than the KPS and SDNN did not meet statistical significance.

## 4. Discussion

Our results demonstrate that the KPS and HRV were independent prognostic factors for OS for BM patients with an age under 65 both by univariate analysis and multivariate analysis. Understanding the prognosis of younger-aged BM patients is of clinical importance. With emerging novel technology, various treatment modalities are available and have been proved to be effective for BM such as surgical resection, stereotactic radiosurgery (SRS), hippocampus-avoidance WBRT, and conventional WBRT. These options could be used alone or in combination, and the treatment choice should be tailored based on expected survival [8]. Previous studies have shown that age is a prognostic factor for survival for BM patients, but few studies have addressed this specific group of non-elderly patients [11]. Although different cutoff values of age have been used, this younger group of patients tended to have longer survival than the elderly [4,5,6,7], and they are more suitable for aggressive treatment or prospective trials. In this study, age of 65 years was used for defining non-elderly because this value was used in the landmark study conducted by Gasper et al. using WBRT for patients with BM [4]. In addition, De Couck et al. have shown that HRV, particularly the index SDNN, derived from 10 s ECG can predict survival in cancer patients age under 65 [22]. Our finding of incorporating HRV analysis before treatment starts for better risk stratification is encouraging. To the best of our knowledge, this is the first study in the literature focusing on non-elderly BM patients with assessment of HRV.

The direct mechanism between low HRV and worse prognosis among BM patients is not yet fully understood; however, understanding the underlying control of HRV provided strong support for this correlation. HRV is regulated predominantly by parasympathetic control via the vagus nerve [12,13], and the function of the vagus nerve is associated with systemic tumor progression and prognosis [16,23]. Gidron et al. postulated the model presenting the vagus nerve as the mediator of tumor-to-brain communication [16]. The vagus nerve transmits signals of peripheral inflammation and oxidative stress, both of which are related to the tumorigenesis microenvironment, to the brain, and it also translates the central control from the brain to reduce proinflammation and oxidative stress in the periphery [24,25]. This implies that for patients with impaired vagal function, the brain may lose its ability to modulate systemic cancer progression, which could lead to worse prognosis. This hypothesis had been supported by previous studies regarding the HRV and survival prognosis among cancer patients [23].

In addition to the tumor-to-brain control via the vagus nerve, impaired cardio-cerebral circulation control could be another factor that leads to worse survival. Patients with lower HRV present with attenuated cardiac vagal activity, which leads to impaired regulation of cerebral circulation [15,26]. In addition, previous studies also demonstrated that subjects with lower HRV had a substantially higher risk of major cardiac events that could lead to sudden or non-sudden cardiovascular death [14,27]. Taking these together, BM patients with lower HRV were at higher risk for subsequent cardio-cerebral circulation accidents that could lead to sudden or non-sudden deaths even without documented systemic tumor progression.

In present study, HRV was assessed using the index of SDNN, which stands for the standard deviation of the interval lengths between each heartbeat. The SDNN is an index generated from time-domain analysis and represents the global HRV [13]. Several previous studies have shown that the SDNN correlates with survival among cancer patients and different cutoff values have been used among different clinical scenarios [19,21,28,29,30]. Here, we chose an SDNN of 10 ms for survival analysis based on previous results of patients with BM [19]. However, as this study focused on a relatively young age of the population, we tested whether a correlation between the SDNN and each factor existed. In addition, multivariate analysis was also conducted to confirm that the SDNN is an independent prognostic factor.

The assessment of HRV is simply from ECG recordings. As ECG is a non-invasive tool, it is good for the clinical setting to evaluate BM patients. However, factors that could influence HRV exist. For instance, medications such as beta-blockers or anti-arrhythmic drugs, the length of ECG recordings for analysis [13], and previous mediastinal irradiation [31] have all been reported. Thus, as a prospective study focusing on the use of HRV, a standardized protocol was used to minimize possible confounding factors for HRV assessment. In addition, 5-min ECGs were recorded and used for analysis as we thought it was more suitable than 24-h recording for radiation oncology outpatient setting and for BM patients.

Performance status has long been used as a survival prognostic factor for BM patients [4,5,6,7]. In addition, a retrospective study conducted by Nieder et al. [11] also showed significant survival difference with KPS among young age BM patients by means of univariate analysis. Our results further confirm these findings. In the present study, we tested the prognostic significance of performance status not only by means of univariate analysis but also using multivariate analysis. Patients with a KPS of 80 or more had significant longer survival comparing to those with a KPS < 80, with a hazard ratio of 3.06. Though different values were used by different researchers, evaluating the KPS for non-elderly BM patients is still a powerful tool for risk stratification.

Although patients in this study were of a relatively younger age, the treatment that we used was uniformly WBRT. Needless to say, this treatment could lead to significant neurocognitive impairment [32,33,34]. The rationale for using WBRT in this study was the reimbursement policy of our National Health Insurance, and it reflects the real-world practice here in our country. According to our reimbursement policy, precise and less harmful treatment such as SRS could only be considered for BM patients with a good performance status, controlled primary tumors, without extracranial metastases, fewer than four intracranial lesions, and lesions with diameter smaller than 3 cm [35], as all these factors together predict longer expected survival [4,5,6,7]. In this study cohort, with all patients being aged under 65 years, only one patient met all these reimbursement criteria, except for the fact that she was diagnosed with multiple intracranial lesions, meaning that the application of SRS was not suitable. Our study was not aimed at testing different treatment methods; however, based on our results, we would suggest that the choice of treatment options for patients with a good KPS and higher HRV should be considered differently, especially for these younger-aged BM patients.

There are several limitations in this study. First, the treatment was uniformly WBRT, and BM patients treated by using other modalities such as surgical resection or SRS were not included. Second, only one patient in this cohort met the criterion of RPA I, meaning that whether our findings can be applied to this group remains unclear. Third, the primary origins of BM were heterogenous, and thus disease-specific outcomes require further investigation.

## 5. Conclusions

The outcomes of non-elderly BM patients treated using WBRT were studied. The results of our small series prospective cohort reveal that the HRV index SDNN, in addition to the KPS, is an independent survival prognostic factor. The risk stratification of life expectancy with the incorporation of HRV is encouraging. Prospective studies based on these results for further investigation are warranted.

## Figures and Tables

**Table 1:** Demographic data of all non-elderly brain metastasis patients.

Parameter	*n*	%
Gender		
Female	24	60.0%
Male	16	40.0%
Age		
<40	3	7.5%
40–49	6	15.0%
50–59	17	42.5%
60–64	14	35.0%
KPS		
40–50	9	22.5%
60–70	17	42.5%
80–90	14	35.0%
Extracranial metastases		
Without	8	20.0%
With	32	80.0%
Primary Status		
Not controlled	35	87.5%
Controlled	5	12.5%
Number of Metastasis		
1	4	10.0%
2–3	6	15.0%
>3	30	75.0%
Volume of the largest lesion		
<5 cm^3^	23	57.5%
5–13 cm^3^	9	22.5%
≥13 cm^3^	8	20.0%
Primary		
NSCLC	24	60.0%
SCLC	3	7.5%
Breast	7	17.5%
Others	6	15.0%
DM		
With	6	15.0%
Without	34	85.0%
HTN		
With	10	25.0%
Without	30	75.0%
SDNN		
<10 ms	13	32.5%
≥10 ms	27	67.5%
RPA Class		
I	1	2.5%
II	28	70.0%
III	11	27.5%

Abbreviations: KPS: Karnofsky performance status; NSCLC: non-small cell lung cancer; SCLC: small cell lung cancer; DM: diabetes mellitus; HTN: hypertension; SDNN: standard deviation of normal-to-normal intervals.

**Table 2:** Comparison of parameters between SDNN groups.

	SDNN < 10 ms	SDNN ≥ 10 ms	*p* Value
Age	57.23 ± 6.60	52.52 ± 8.56	0.089
Gender			
Female	6	18	0.305
Male	7	9	
KPS			
<80	10	16	0.316
≥80	3	11	
Extracranial metastases			
Without	2	6	1.000
With	11	21	
Primary status			
Not controlled	11	24	1.000
Controlled	2	3	
Number of metastases			
≤3	4	6	0.700
>3	9	21	
Volume of the largest lesion			
≤5 cm^3^	6	17	0.496
>5 cm^3^	7	10	
Primary			
NSCLC	8	16	1.000
Others	5	11	
DM			
With	1	5	0.643
Without	12	22	
HTN			
With	5	5	0.246
Without	8	22	

Abbreviations: SDNN: standard deviation of normal-to-normal intervals; KPS: Karnofsky performance status; NSCLC: non-small cell lung cancer; SCLC: small cell lung cancer; DM: diabetes mellitus; HTN: hypertension.

**Table 3:** Univariate analysis on overall survival.

Parameters	*n*	Overall Survival			
		Median (months)	3M	6M	*p* Value
All Participant	40	6.21	62.5%	52.5%	-
Gender					
Female	24	7.63	66.7%	54.2%	0.420
Male	16	3.62	56.3%	50.0%	
KPS					
<80	26	2.17	42.3%	34.6%	0.019
≥80	14	9.93	100.0%	85.7%	
Extracranial metastases					
Without	8	12.07	87.5%	87.5%	0.245
With	32	3.91	56.3%	43.8%	
Primary status					
Not controlled	35	6.21	60.0%	51.4%	0.196
Controlled	5	21.47	80.0%	60.0%	
Number of Metastases					
≤3	10	6.21	70.0%	60.0%	0.493
>3	30	5.65	60.0%	50.0%	
Volume of the largest lesion					
≤5 cm^3^	23	8.52	65.2%	56.5%	0.949
>5 cm^3^	17	4.47	58.8%	47.1%	
Primary					
NSCLC	24	8.02	62.5%	58.3%	0.312
Others	16	3.91	62.5%	43.8%	
DM					
With	6	12.92	83.3%	83.3%	0.515
Without	34	4.47	58.8%	47.1%	
HTN					
With	10	7.63	60.0%	60.0%	0.463
Without	30	5.65	63.3%	50.0%	
SDNN					
<10 ms	13	2.70	46.2%	30.8%	0.007
≥10 ms	27	8.88	70.4%	63.0%	

Abbreviations: KPS: Karnofsky performance status; NSCLC: non-small cell lung cancer; DM: diabetes mellitus; HTN: hypertension; SDNN: standard deviation of normal-to-normal intervals.

**Table 4:** Multivariate analysis on overall survival.

Parameters	Overall Survival		
	*p* Value	HR	95% CI
**KPS**: <80 vs. ≥80	0.018	2.349	1.159–4.762
**SDNN**: <10 ms vs. ≥10 ms	0.008	2.766	1.306–5.857

Abbreviations: HR: hazard ratio; CI: confidence interval; KPS: Karnofsky performance status; SDNN: standard deviation of normal-to-normal intervals.

## Data Availability

The data presented in this study are available on request from the corresponding author. The data are not publicly available due to the nature of this research, participants of this study did not agree for their data to be shared publicly.
